# Coexistence of Orthostatic Hypotension and Refractory Hypertension in Diabetic Cardiovascular Autonomic Neuropathy: A Therapeutic Dilemma

**DOI:** 10.7759/cureus.110358

**Published:** 2026-06-06

**Authors:** Zaid Qadri, Roshini Kadimcherla, Binuja A Sam, Eleanor Reffin, Tawhida Youshra, Tamer Mikhail

**Affiliations:** 1 Acute Medicine, King's College Hospital NHS Foundation Trust, London, GBR; 2 General Medicine, King's College Hospital NHS Foundation Trust, London, GBR; 3 Internal Medicine, King's College Hospital NHS Foundation Trust, London, GBR; 4 Internal Medicine, Lewisham and Greenwich NHS Trust, London, GBR; 5 Medicine, King's College Hospital NHS Foundation Trust, London, GBR; 6 Internal Medicine, Princess Royal University Hospital, London, GBR

**Keywords:** baroreceptor reflex, diabetic cardiovascular autonomic neuropathy, microvascular complications of diabetes, orthostatic hypotension, refractory hypertension

## Abstract

Diabetic cardiovascular autonomic neuropathy (DCAN) is a common, but underdiagnosed, complication of diabetes. The condition arises due to damage to the autonomic nerve fibres innervating the heart and vasculature. This can manifest clinically in a variety of ways, including coexisting orthostatic hypotension and supine hypertension. We present a case of DCAN in a middle-aged patient, which highlights how debilitating this condition can be and the therapeutic challenge posed to clinicians by its management. This female patient had severe orthostatic hypotension, causing recurrent collapses and limited mobility, alongside refractory hypertension, which progressed to cause target organ damage. Her management required a multidisciplinary approach including medical (cardiology, renal, neurology, endocrinology), physiotherapy, and occupational therapy teams. Her medication regimen was frequently titrated and modified to try to find a balance in blood pressure that maintained safety and functional status. Ultimately, she required a combination of five antihypertensive medications, and the orthostatic hypotension could only be managed supportively. This did not provide a satisfactory functional outcome for the patient, whose mobility and independence were severely restricted by haemodynamic fluctuations. This calls attention to the need for further research into management strategies for this condition.

## Introduction

Orthostatic hypotension and supine hypertension share a complex connection through the physiology of blood pressure (BP) regulation. On standing, there is a drop in BP due to venous pooling. During a normal physiological response, baroreceptors in the carotid sinus and the aortic arch detect this and respond by increasing sympathetic tone and decreasing parasympathetic tone. The influx of sympathetic tone results in increased peripheral vascular resistance, leading to an increase in venous return and maintained BP. The withdrawal of parasympathetic tone results in the augmentation of heart rate. Orthostatic hypotension is defined as a sustained reduction of systolic BP of at least 20 mmHg or diastolic BP of 10 mmHg, within three minutes of standing or on tilting the body to at least 60 degrees on a tilt table [1]. This results from an insufficient physiological response to the decreased venous return upon standing [2].

Supine hypertension is defined by the American Autonomic Society and the European Federation of Autonomic Societies as a systolic BP of ≥140 mmHg and a diastolic BP of ≥90 mmHg while in the supine position. Paradoxically, supine hypertension is physiologically linked to orthostatic hypotension [2]. Episodes of orthostatic hypotension during the day activate the renin-angiotensin-aldosterone pathway, which regulates BP, exacerbating supine hypertension. Meanwhile, supine hypertension causes significant nocturnal diuresis, which can worsen daytime orthostatic hypotension [2,3].

In this case, we describe a patient with orthostatic hypotension that coexisted with supine hypertension. The BP dysregulation was attributed to diabetic cardiovascular autonomic neuropathy (DCAN), one of the least understood microvascular complications of diabetes. Clinical manifestations of DCAN include resting heart rate disorders, silent myocardial ischemia, QTc prolongation, and orthostatic alterations in heart rate and BP. These occur secondary to damage to cardiovascular autonomic nerves, resulting in a dysregulated sympathovagal balance [3].

This patient had developed DCAN (and other complications) secondary to a late diagnosis of diabetes. Her hypertension became resistant and eventually refractory, with target organ damage. Refractory hypertension is defined as BP that is uncontrolled despite using ≥5 antihypertensive medications of different classes, including a long-acting thiazide diuretic and a mineralocorticoid receptor antagonist (MRA) at maximal or maximally tolerated doses. This phenotype is different from resistant hypertension, BP that is uncontrolled with ≥3 medications, with common regimens usually including a long-acting calcium channel blocker, a blocker of the renin-angiotensin system, and a diuretic [4]. Refractory hypertension is in itself a challenge to manage, but attempts must be made so as to minimise target organ damage. When combined with debilitating orthostatic drops, optimal BP control can therefore be very difficult. 

## Case presentation

A 57-year-old woman was admitted in July 2025 following an unwitnessed fall. This was her sixth admission in 10 months, with all hospitalisations being for recurrent collapses, severe orthostatic hypotension, and resistant hypertension.

The timeline below outlines the events from her initial diagnosis of diabetes to her most recent admission, summarising the progression of her condition.

Clinical timeline

In May 2023, the patient presented with acute anterior uveitis, during which incidental hyperglycaemia (blood glucose (BG): 21.8 mmol/L; normal value: <5.6 mmol/L) and mild diabetic retinopathy were identified. This led to further diabetic assessment, and by January 2024, HbA1c was 117 mmol/mol (normal value: <42 mmol/mol), confirming uncontrolled type 2 diabetes mellitus (T2DM) with diabetic retinopathy, for which metformin and atorvastatin were initiated. Despite treatment, the patient subsequently experienced her first collapse in June 2024, resulting in a traumatic brain injury. Investigations at that time revealed 50% right internal carotid artery (R ICA) stenosis on carotid Doppler and impaired diastolic function on echocardiography. Following a second collapse, the patient was admitted between September and October 2024, where orthostatic hypotension and chronic kidney disease (CKD) stage 3 were identified. Further investigations ruled out hypertrophic cardiomyopathy (HOCM) and adrenal insufficiency through cardiac magnetic resonance imaging (MRI) and a short synacthen test, respectively. A loop recorder was inserted, and treatment with fludrocortisone and midodrine was commenced. However, symptoms persisted, leading to a third collapse and a second admission between November 2024 and March 2025. During this period, the patient developed gastroparesis alongside persistent orthostatic hypotension. Autonomic screening tests confirmed fairly well-established cardiovascular autonomic failure, while ganglionic ACh receptor antibody testing was negative. Consequently, pyridostigmine was introduced, and a package of care was arranged. Nevertheless, the patient was readmitted between March and April 2025 with dizziness, syncope, chronic diarrhoea, and hypertension, prompting the initiation of amlodipine and the temporary cessation of pyridostigmine. The clinical course then became increasingly complicated, and during a fourth admission in May 2025, the patient developed decompensated heart failure with preserved ejection fraction (HFpEF) and CKD stage 3. Echocardiography demonstrated moderate concentric left ventricular hypertrophy, leading to treatment with furosemide, later switched to bumetanide, alongside adjustment of fludrocortisone therapy. Shortly afterwards, between May and June 2025, the patient required a fifth admission due to hypertensive crisis and pulmonary oedema. Repeat autonomic testing continued to demonstrate cardiovascular autonomic abnormalities, resulting in the initiation of dapagliflozin and darbepoetin, with further adjustments to antihypertensive therapy. Despite these interventions, the patient's condition continued to deteriorate, culminating in a sixth admission between July and October 2025 following a fall. During this admission, the patient developed acute kidney injury (AKI) on a background of CKD and refractory hypertension. Orthostatic hypotension medications were discontinued, and following multidisciplinary autonomic review, it was concluded that therapeutic options were limited, with the patient ultimately becoming wheelchair-dependent.

Investigations

Investigations were undertaken to identify the potential causes of the patient's progressive symptoms and recurrent collapses. Renal assessment demonstrated a raised albumin-to-creatinine ratio and reduced estimated glomerular filtration rate (eGFR), leading to a new diagnosis of CKD, which was considered multifactorial in origin, likely resulting from longstanding hypertension and possible diabetic nephropathy. Cardiac imaging included a cardiac MRI, which showed left ventricular hypertrophy without evidence of other structural cardiac abnormalities. Transthoracic echocardiography further demonstrated moderate concentric left ventricular hypertrophy with preserved systolic function and an ejection fraction of 60-65%. An extensive endocrine and autoimmune workup was performed to exclude the secondary causes of autonomic dysfunction. Serum cortisol levels were elevated, consistent with a physiological stress response, while thyroid function tests (thyroid-stimulating hormone (TSH) and free T4) were within normal limits. Autoimmune screening was unremarkable, with negative antinuclear antibodies (ANA), normal IgM levels, anti-double-stranded DNA (anti-dsDNA) of 0.60, IgA of 3.18, connective tissue disease screen of 0.1, and an elevated kappa light chain level of 33.05 with a normal kappa-to-lambda ratio of 1.58. Computed tomography (CT) of the abdomen showed no adrenal masses, and there was no evidence to support a diagnosis of Addison's disease. Infectious and neoplastic causes were also investigated; full-body CT imaging demonstrated no evidence of malignancy, although benign renal cysts were noted, and inflammatory markers remained within normal limits. Nutritional assessment revealed adequate dietary intake, with the patient's weight loss attributed to chronic catabolic illness and progressive deconditioning rather than malnutrition. In view of the persistent orthostatic symptoms, a comprehensive autonomic screening assessment was undertaken, the results of which are summarised in Table 1.

**Table 1:** Autonomic screening tests: December 2024 BP: blood pressure

Test	Phase/time point	BP (mmHg)	Heart rate (bpm)	Findings/interpretation
Active standing and sitting recovery test	Supine	204/110	88	Baseline supine hypertension
	Standing 1 min	94/58	97	Marked orthostatic hypotension
	Sitting 2 min	99/72	93	Partial BP recovery
	Sitting 3 min	102/72	91	Persistent hypotension
	Sitting 5 min	116/71	91	Incomplete BP recovery
Isometric exercise	Before exercise	196/101	87	Baseline measurement
	During exercise	199/107	90	Mild hypertensive response
Mental arithmetic	Before test	171/94	88	Baseline measurement
	During test	177/105	91	BP rise demonstrated on beat-to-beat assessment
Cutaneous cold pressor test	Before test	176/104	88	Baseline measurement
	During test	196/107	88	Exaggerated pressor response
Hyperventilation test	Before test	202/104	90	Baseline measurement
	During test	157/92	93	Significant BP reduction
Deep breathing test	During deep breathing	-	-	Respiratory sinus arrhythmia absent
Valsalva manoeuvre	-	-	-	Not performed due to clinical considerations
60° head-up tilt test	Supine	209/111	91	Baseline supine hypertension
	Tilt 1 min	152/85	90	Significant orthostatic BP drop
	Tilt 4 min	91/67	91	Progressive hypotension
	Tilt 7 min	82/58	92	Severe orthostatic hypotension
	Tilt 8 min	83/55	92	Persistent severe hypotension
Plasma catecholamine sampling	-	-	-	Blood sampling not performed due to medication interference
Assessment of foot discolouration	During orthostasis	-	-	Discolouration could not be assessed
Autonomic smell test	-	-	-	Results: 3/12 correct answers. Score falls at or below the 10th percentile for age and is considered clinically abnormal

Clinical course narrative

This patient was initially diagnosed with T2DM during a workup for visual disturbances. The diagnosis was made late in the disease course, with an HbA1C of 117 mmol/mol (normal value: <42 mmol/mol), and her diabetes had progressed to microvascular complications (diabetic retinopathy and CKD). Within six months of diagnosis, she began to experience frequent presyncopal and syncopal episodes, associated with orthostatic drops in her systolic BP of >40-60 mmHg. In her first three admissions, she received maximal therapy for orthostatic hypotension: fludrocortisone, midodrine, compression stockings, abdominal binders, and a trial of pyridostigmine. Despite these measures, she continued to experience collapses. Extensive investigations ruled out other causes, such as arrhythmia, and her symptoms were concluded to be secondary to DCAN. 

**Figure 1:**
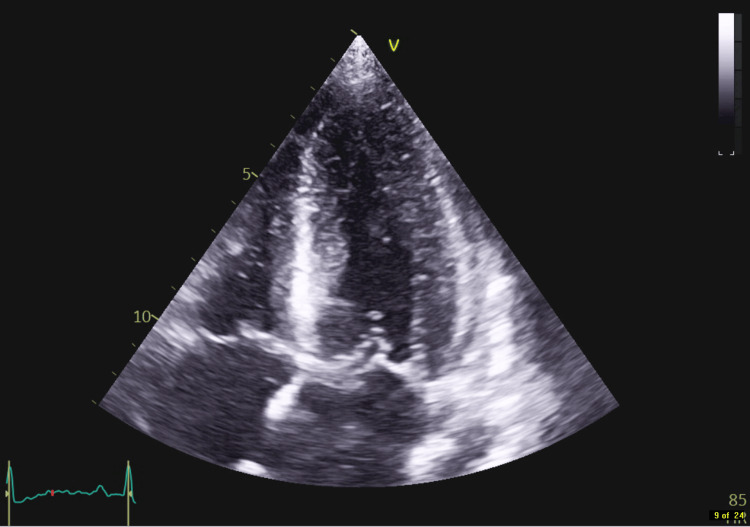
Apical view of the heart showing hypertrophy of the left ventricle

**Figure 2:**
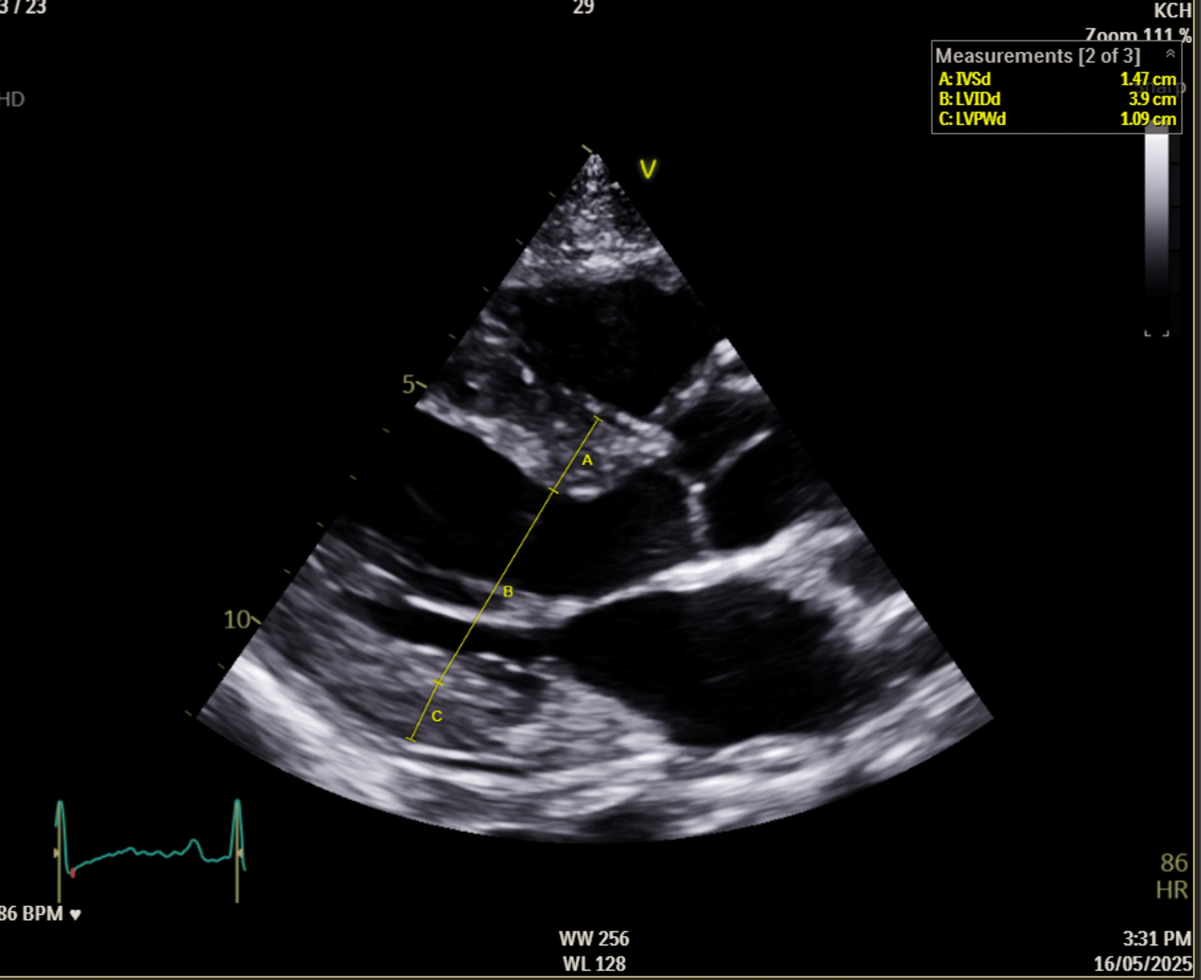
Parasternal long axis view with measurement of IVSD, LVIDD, and LVPWD IVSD: interventricular septal diameter; LVIDD: left ventricular internal diameter; LVPWD: left ventricular posterior wall diameter

Between the episodes of collapse, this patient was also noted to have elevated BP. The coexisting supine hypertension was first managed with medication in her third admission, but became a more significant issue during the next two. First, an echocardiogram showed left ventricular hypertrophy, likely secondary to hypertension, which can be seen in Figures 1-2. Then, she presented with a hypertensive crisis and pulmonary oedema, and the real dilemma of management started. Treating the hypertension, while balancing management for symptomatic orthostatic hypotension, posed a significant clinical challenge. This situation was further complicated by coexisting AKI on CKD, electrolyte abnormalities, gastroparesis, and anaemia of chronic disease.

Table 2 shows the changes in her medications made through her six admissions, in an attempt to manage her BP fluctuations.

**Table 2:** Changes in the patient's medications made through her six admissions OD: once a day; BD: twice a day; TDS: three times a day

	1st admission	2nd admission	3rd admission	4th admission	5th admission	6th admission
Fludrocortisone	300 mcg OD	200 mcg OD	200 mcg BD	50 mcg BD	50 mcg BD	Stopped
Midodrine	5 mg TDS	10 mg TDS	10 mg TDS	10 mg TDS	10 mg BD	Stopped
Pyridostigmine bromide	-	30 mg BD	Stopped	-	-	-
Amlodipine	-	-	10 mg OD	10 mg OD	Stopped	-
Furosemide	-	-	-	20 mg OD	Stopped	-
Doxazocin	-	-	-	-	2 mg OD	Stopped
Bumetanide	-	-	-	-	1 mg BD	Stopped
Bisoprolol	2.5 mg BD --> stopped	-	-	-	-	3.75 mg OD
Clonidine	-	-	-	-	-	100 mcg TDS
Hydralazine	-	-	-	-	-	50 mg QDS
Irbesartan	-	-	-	-	-	300 mg OD
Indapamide	-	-	-	-	-	1.5 mg OD
Spironolactone	-	-	-	-	-	25 mg OD --> stopped
Ramipril	-	-	-	-	-	5-10 mg --> stopped

Over the span of a year, this patient spent prolonged periods of time in the hospital, with a maximum of only 20 consecutive days at home. Management required frequent medication adjustments under multidisciplinary input, including cardiology, renal, neurology, endocrinology, autonomic, and vascular teams. Antihypertensives trialled included amlodipine, ramipril, doxazosin, irbesartan, hydralazine, clonidine, bisoprolol, indapamide, and spironolactone. Orthostatic hypotension medications were titrated and altered, but finally stopped, as addressing the refractory hypertension took priority. The final regimen of antihypertensives included a complex combination of irbesartan, hydralazine, clonidine, indapamide, and bisoprolol. The non-pharmaceutical measures included use of abdominal binders and compression stockings, exercises to maintain good leg and core muscle tone, equipment such as a perching stool, grab rails, and a rollator with a seat, and practical measures such as keeping shower temperature cool and spraying her feet with cold water if she feels hypotensive to enable vasoconstriction of the pooled venous blood. However, persistent haemodynamic instability combined with deconditioning from prolonged admission led to a decline in mobility. At the point of discharge, this patient could only mobilise with a wheelchair and required significant ongoing community support.

## Discussion

Cardiac autonomic neuropathy (CAN) is one of the most underdiagnosed complications of diabetes mellitus. The prevalence of CAN varies between 1% and 90% in patients with type 1 diabetes mellitus (T1DM) and 20% and 73% in patients with T2DM. This huge variation in CAN prevalence is due to the inconsistency in the criteria used to diagnose CAN and significant differences in the study populations, particularly in relation to CAN risk factors (such as age, gender, and diabetes mellitus duration among others). Early recognition is crucial as CAN is detected in about 7% of both T1DM and T2DM at the time of initial diagnosis, and it is estimated that the risk for developing CAN increases annually by approximately 6% and 2% in patients with T1DM and T2DM, respectively. Ethnicity has also been postulated to be a risk factor for CAN as South Asians seem to have lower rates of peripheral neuropathy than White Europeans with diabetes mellitus [1].

As evidenced by this case, it is essential to be aware of DCAN and maintain a high index of suspicion in patients presenting with poor glycaemic control or cardiovascular risk and labile BP. Diagnostic tests for DCAN include cardiovascular autonomic reflex tests (CARTs), such as heart rate variability, orthostatic hypotension test, sustained handgrip test, and myocardial scintigraphy [5]. Once a diagnosis has been made, the challenge of DCAN lies in managing BP extremes and improving functional status. When orthostatic hypotension and supine hypertension coexist, these conditions tend to exacerbate each other through the homeostatic mechanisms of BP control. Furthermore, both extremes pose a risk to the patient. Orthostatic hypotension predisposes patients to syncope and falls. Hypertension contributes to worsening renal function, progression of cardiovascular disease (such as left ventricular hypertrophy), and increased all-cause mortality. Current recommendations emphasise the management of orthostatic hypotension as a priority over aggressive BP control in the elderly population; however, no guidelines exist for younger patients [6].

Ideally, therapies for patients with orthostatic hypotension and supine hypertension would selectively improve upright BP without increasing supine BP. Unfortunately, most pharmacological agents used in orthostatic hypotension increase both supine and upright BPs. The two most commonly used medications, fludrocortisone and midodrine, in fact increase supine BP more than upright BP [6]. The patient underwent trials of maximum medical therapy, including fludrocortisone, midodrine, and pyridostigmine. Despite these interventions, her orthostatic symptoms persisted and progressively impaired her mobility. Concurrently, she developed worsening supine hypertension, resulting in target organ damage. Given the unfavourable risk-benefit profile, all her orthostatic hypotension meds were subsequently discontinued. 

The management of supine hypertension also poses a challenge. During the daytime, it can usually be managed by avoiding lying flat; however, nighttime hypertension is more problematic. Most of the patients with orthostatic hypotension show little or no nighttime BP drop ("nondipping" or "reverse dipping") and experience significant nighttime urine and sodium loss, which worsens morning orthostatic hypotension [7]. Furthermore, antihypertensives themselves pose a risk of increased hypotensive episodes, especially in the morning. The treatment goal is to selectively lower nighttime BP, reduce this natriuresis, and improve morning BP. Short-acting antihypertensives given before bed may help, and possible agents include nitroglycerin, sildenafil, clonidine, nebivolol, nifedipine, losartan, or eplerenone. Among these, only losartan decreases nighttime sodium loss. Unfortunately, none of the agents are shown to improve daytime orthostatic hypotension [6].

**Figure 3:**
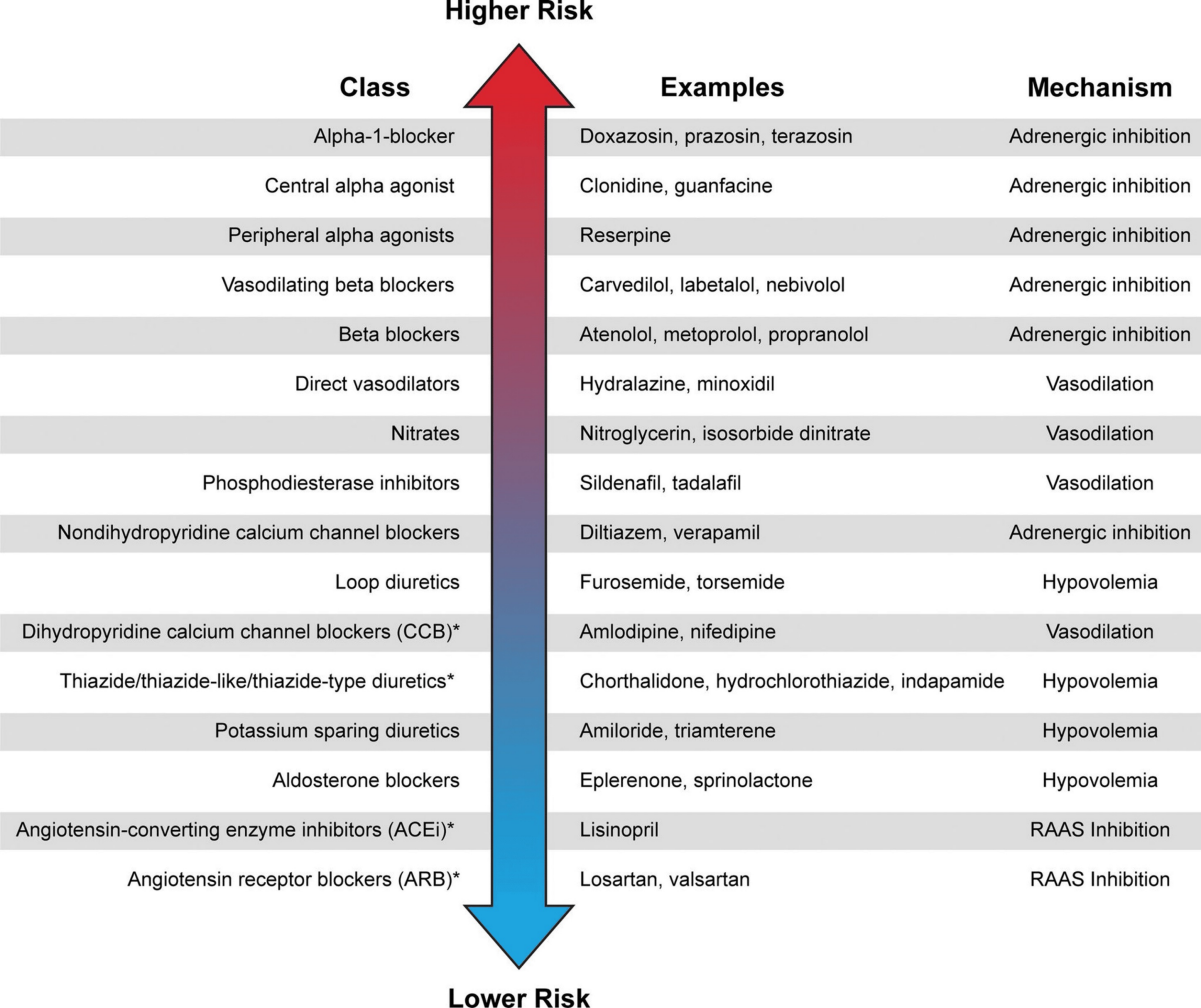
Relative ranking of hypertension classes according to risk of orthostatic hypertension * denotes a class considered first-line treatment for hypertension.

Figure 3, provided by the American Heart Association, provides a guideline on which antihypertensives are comparatively safer to use for trial in patients with orthostatic hypotension and resistant hypertension [6].

Pharmacological management must be handled carefully in patients with autonomic dysfunction. It is advisable to start with monotherapy and monitor the response before adding further medications. Patient comorbidities must always be considered. In this patient, the choice of antihypertensive agent was further limited by poor kidney function, gastroparesis, and cardiac issues. Centrally acting antihypertensives (such as alpha and beta blockers) were gradually stopped as they worsened her postural drops. Short-acting antihypertensive agents such as clonidine, irbesartan, and hydralazine were tried, with doses adjusted according to the clinical response. The medications best suited for patients with coexisting hypertension (aldosterone antagonists like spironolactone and angiotensin-converting enzyme (ACE) inhibitors such as ramipril) were tried, but had to be stopped due to the development of dangerous electrolyte imbalances on the background of CKD. Multiple further combinations were trialled while taking into account medication side effects and existing comorbidities; however, BP control remained suboptimal. Eventually, her supine hypertension progressed to refractory hypertension, with evidence of target organ damage in the form of left ventricular hypertrophy.

The management of DCAN requires a multidisciplinary approach from early in the disease course. These patients are usually complex, and input from cardiology, endocrinology, and clinical pharmacology can be valuable. Physiotherapy and occupational therapy teams must be involved to address the patient's mobility requirements and prevent deconditioning. They can also advise on the use of abdominal binders and compression stockings. As in this case, medical management may ultimately prove unsuccessful in stabilising BP fluctuations to a satisfactory degree. It is therefore ultimately these teams who prove essential to maximising patient function and independence on discharge. 

## Conclusions

This case highlights the severe functional burden of DCAN and the therapeutic challenge posed by coexisting orthostatic hypotension and supine hypertension. Despite coordinated multidisciplinary care and prolonged inpatient management, sustained haemodynamic stability and meaningful functional recovery were not achieved.

Clinicians should consider DCAN in patients with long-standing or poorly controlled diabetes presenting with labile BP and recurrent syncope as advanced autonomic failure markedly limits treatment response in patients with delayed diagnosis.

Management remains largely empirical, requiring iterative adjustment of pressor agents for orthostatic hypotension and antihypertensives for supine hypertension, alongside non-pharmacological measures such as compression therapy, head-up sleeping, and volume optimisation. However, agents including midodrine, fludrocortisone, pyridostigmine, and short-acting nocturnal antihypertensives often provide incomplete or conflicting control.

There is a need for improved strategies targeting autonomic dysfunction, including sympathetic modulation, baroreflex activation therapy, and chronotherapy approaches, alongside structured multidisciplinary care pathways.

Further research is urgently needed into mechanism-based therapies that stabilise upright BP without worsening supine hypertension, as current approaches frequently fail to achieve sustained haemodynamic balance.
